# Acute Embolic Events –Myocardial Infarction and Stroke, in the Presence of an Interatrial Septal Aneurysm

**Published:** 2011-08-25

**Authors:** CD Calin, A Calin, M Lupu, A Bucsa, C Ginghina

**Affiliations:** ‘Prof. Dr. C. C. Iliescu’ Emergency Institute of Cardiovascular Diseases, Bucharest Romania

**Keywords:** paradoxical embolism, patent foramen ovale, myocardial infarction, stroke

## Abstract

We will present the case of a 52 year old patient, admitted to our Department for exertional angina, with 2 recent acute events – inferior myocardial infarction and stroke. The coronary angiography revealed patent coronary arteries, without atherosclerotic lesions. The transthoracic echocardiography established the presence of an interatrial septal aneurysm with interatrial shunt. Under these circumstances, we have considered the presence of paradoxical embolism as a potential pathophysiological mechanism of the acute ischemic events. The percutaneous closure of the interatrial shunt to prevent the recurrence of embolic events will be discussed.

## Case Report

A 52 year old patient presented to our Department for angina on exertion. He had a familial history of coronary heart disease, was a current smoker and had a poorly controlled arterial hypertension and severe dyslipidemia. From his medical history we mention the presence of an inferior posterior myocardial infarction that was conservatively treated (without a coronary angiography performed at that time) and an ischemic stroke 3 months after the acute coronary event. The patient was admitted to a neurology department in Milan, and the diagnosis was suggested by clinical features: the sudden occurrence of right brachial paresthesias and motor deficit with spontaneous complete recovery within 2 hours and confirmed by imagistic investigations. The cerebral computer tomography scan did not reveal any focal acute lesions but the cerebral MRI had marked out an area of hyposignal at the level of the right cerebral hemisphere, suggestive of an acute ischemic lesion. The patient was discharged after 6 days and went to an ambulatory cardiology department, where an echocardiography was performed and revealed a normally sized left ventricle with inferior and posterior segmental wall motion anomaly and preserved systolic function (LVEF 50% by Simpson's biplane method). No valvular lesions or a cardiac source of embolism were mentioned. The patient also underwent an ECG stress test that was negative for ischemia. 

**Figure 1:**
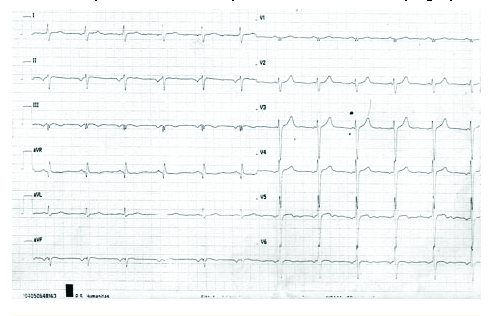
Standard 12 leads electrocardiography–negative P waves in II, III, aVF, left anterior fascicular block, slow progression of the R wave in V1– V3, biphasic, flattened T waves in III, aVF, V4–V6.

**Figure 2:**
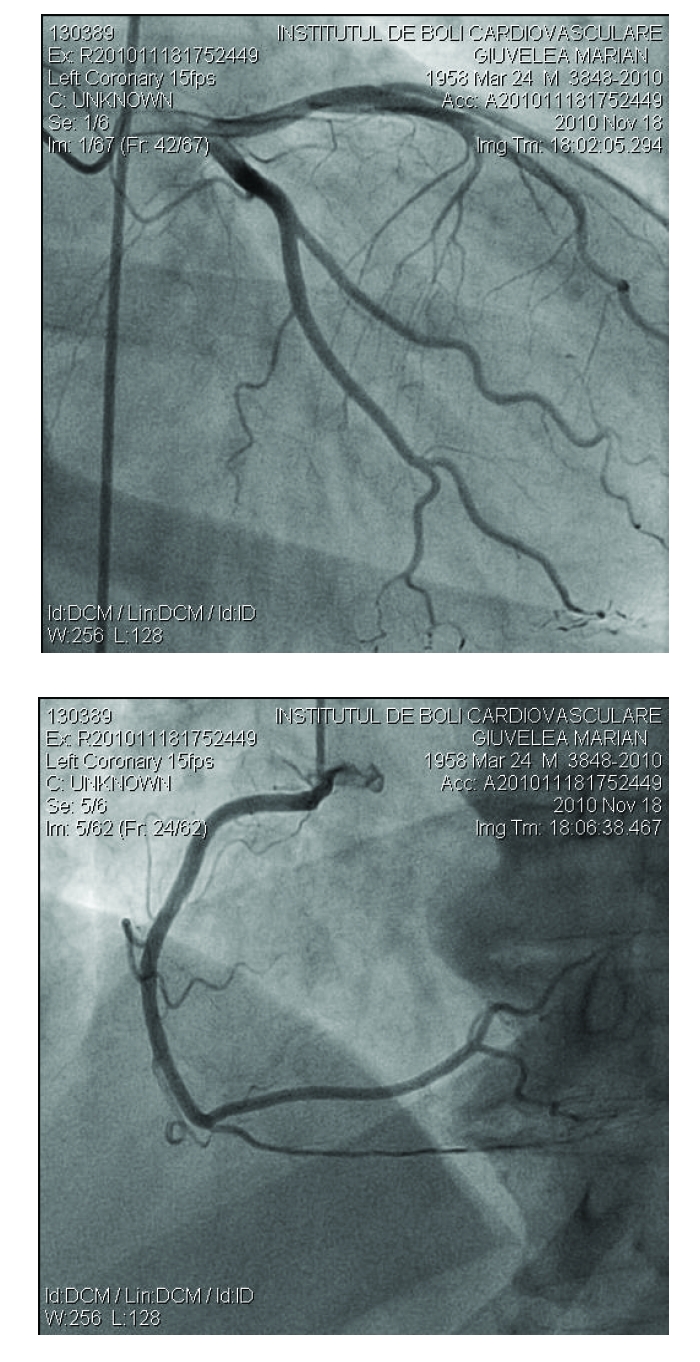
Patent coronary arteries, without evidence of atherosclerotic lesions. Right anterior oblique views of the left coronary artery (left). Left anterior oblique views of the right coronary artery (right).

On admission to our clinic, the ECG showed negative P waves in II, III, aVF suggestive of an atrial ectopic rhythm, left anterior fascicular block, a slow progression of the R wave in V1 –V3 and signs of inferior and lateral ischemia (biphasic, flattened T waves in III, aVF, V4–V6) (Figure 1). In view of the above mentioned medical history and the important cardiovascular risk factors we considered that atherosclerotic coronary lesions might be responsible for the recent coronary event and the recurrent angina in our patient and decided to perform a coronary angiography. To our surprise, no coronary lesions were found (Figure 2).

**Figure 3:**
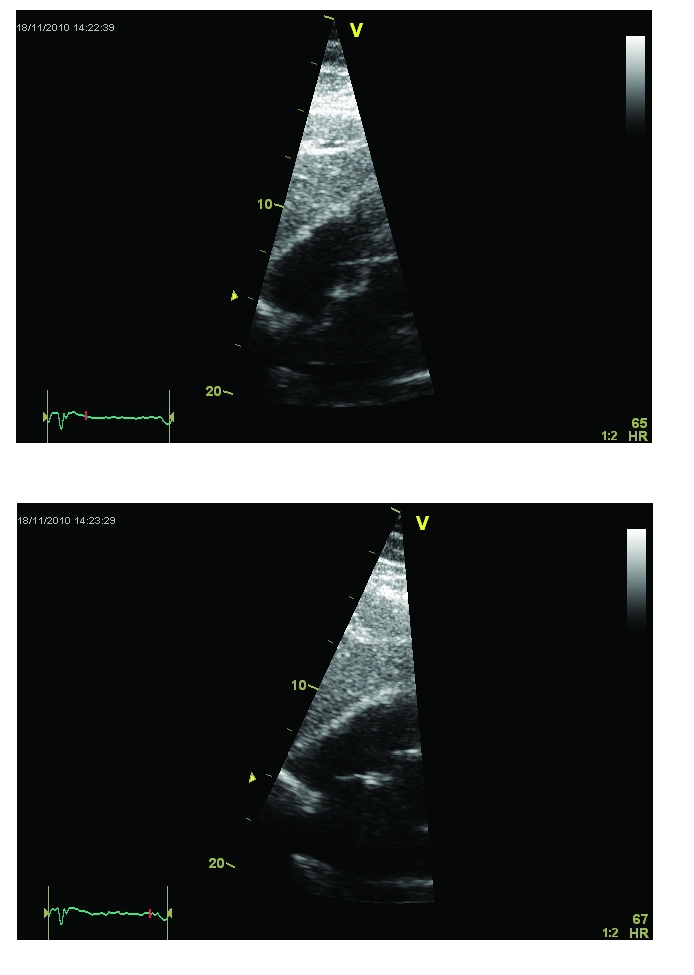
Bidimensional transthoracic echocardiography from the subcostal view. Systolic (A) and diastolic (B) frames showing the ample excursion of the interatrial septum, suggesting the presence of a septal aneurysm.

**Figure 4:**
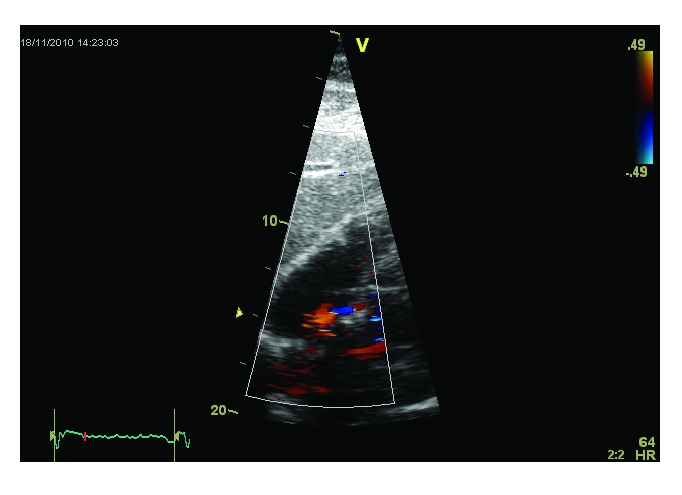
Transthoracic echocardiography, subcostal view: a small interatrial left to right shunt was suggested by the presence of a turbulent flow in color Doppler examination at the level of the interatrial septal aneurysm

The Doppler ultrasound scan of the carotid arteries did not reveal any atherosclerotic lesions. The transthoracic echocardiography indicated a mild global left ventricular systolic dysfunction (LVEF 45–50%) with hypokinesia of the basal inferior wall and posterior interventricular septum, impaired relaxation of the left ventricle and the presence of an interatrial septal aneurysm. The transthoracic contrast echo examination confirmed the presence of an interatrial shunt (Figure 3–Figure 4). 

Lab test findings were within normal limits, except for the persistence of a mild hypercholesterolemia. The patient was tested for the presence of the most frequent procoagulant states – antithrombin III, protein C and S, prothrombin time, partial thromboplastin time, fibrinogen, homocysteine, cardiolipin antibodies and factor V Leiden were all within normal limits.

**Figure 5:**
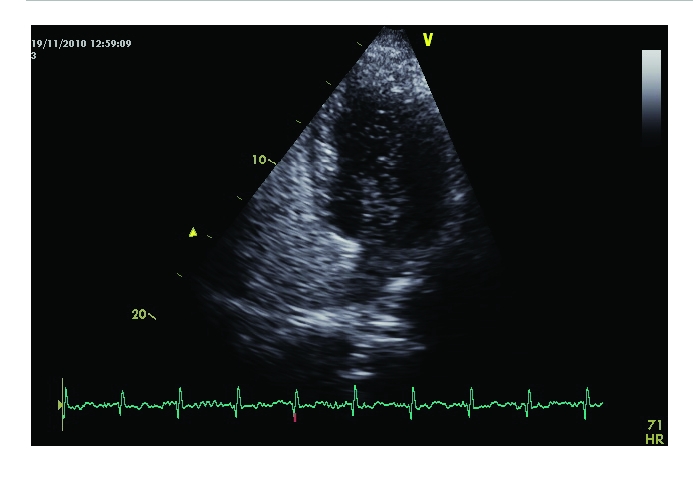
Transthoracic echocardiography, apical 4 chamber view. Immediately after the injection of agitated saline, the contrast is passing from the right atrium to the left atrium, confirming the presence of an interatrial shunt. The contrast is readily seen at the level of the left atrium and passing into the left ventricle.

## Discussion

In patients with cardiovascular risk factors the most frequent cause of myocardial infarction is atherosclerotic coronary artery disease. Paradoxical embolism in the presence of an interatrial septal defect or patent foramen ovale is accepted as a possible mechanism of transient ischemic attack/ischemic stroke and peripheral vascular embolism.[1] Paradoxical emboli at the level of the coronary arteries could lead to acute myocardial infarction with normal epicardial coronary arteries. On the basis of limited pathologic and clinical series, it appears that paradoxical coronary emboli account for 5%–10% of all paradoxical emboli.[2]

In spite of the fact that our patient had cardiovascular risk factors (smoking, hypertension, dyslipidemia, gender, age), the epicardial coronary arteries were free of atherosclerotic lesions. The hypothesis of a procoagulant state in a young patient with prior myocardial infarction and patent coronary arteries was taken into account, but blood tests were negative.[3] Because the acute coronary event happened almost 9 months prior to coronary angiography, no occlusive coronary thrombi (typical for an embolic mechanism) were revealed. In these cases, an accurate diagnosis can be established only by visualizing the embolus passing through the interatrial septal defect.[4]

Management of coronary embolism includes immediate reperfusion therapy (thrombolysis or percutaneous intervention) and prevention of recurrence. Acute MI due to emboli have been successfully treated using thrombus aspiration devices. Strategies for prevention of future paradoxical embolic events include lifelong oral anticoagulation and/or surgical or transcutaneous closure of ASDs or PFOs. There are no randomized controlled trials comparing oral anticoagulation with transcutaneous closure of ASDs and PFOs. Transcatheter PFO closure appears safe to protect against recurrent strokes in patients with cryptogenic stroke. Recurrence rates of neurologic events after surgical closure of PFOs are as high as 20% per year. At present, the only unequivocal indication for device closure of PFOs is recurrence of paradoxical embolic events despite therapeutic anticoagulation. Device closure of ASDs appears to have a low recurrence rate of further paradoxical embolic events and may avoid life–long anticoagulation.[5] 

We consider that oral anticoagulation therapy represents the immediate optimal treatment in our case, but percutaneous closure of the interatrial communication remains an option in order to prevent new paradoxical embolic events in this patient.
